# Elderly emergency patients presenting with non-specific complaints: Characteristics and outcomes

**DOI:** 10.1371/journal.pone.0188954

**Published:** 2017-11-30

**Authors:** Joyce J. H. Wachelder, Patricia M. Stassen, Laura P. A. M. Hubens, Steffie H. A. Brouns, Suze L. E. Lambooij, Jeanne P. Dieleman, Harm R. Haak

**Affiliations:** 1 Department of Internal Medicine, Máxima Medical Centre, Eindhoven, the Netherlands; 2 Department of Health Services Research, and CAPHRI School for Public Health and Primary Care, Maastricht, the Netherlands; 3 Department of Internal Medicine, Division of General Medicine, Section Acute Medicine, Maastricht University Medical Centre, Maastricht, the Netherlands; 4 Máxima Medical Centrum Academy, Máxima Medical Centre, Veldhoven, the Netherlands; Karolinska Institutet, SWEDEN

## Abstract

**Background:**

Non-specific complaints (NSC) are common at the emergency department, but only a few studies have shown evidence that these complaints are associated with a poor prognosis in elderly emergency patients.

**Objective:**

To describe patient characteristics and outcomes in a cohort of elderly emergency patients presenting with NSC. Outcomes were: patient characteristics, hospitalization, 90-day ED-return visits, and 30-day mortality.

**Method:**

A retrospective cohort study was conducted amongst elderly patients present to the Internal Medicine Emergency Department (ED) between 01-09-2010 and 31-08-2011. NSC were defined as indefinable complaints that lack a pre-differential diagnosis needed to initiate of a standardized patient evaluation. Cox regression was performed to calculate Hazard Ratios (HR) and corrected for confounders such as comorbidity.

**Results:**

In total, 1784 patients were enrolled; 244 (13.7%) presented with NSC. Compared to those with SC, comorbidity was higher in the NSC-group (Charlson comorbidity index 3.0 vs. 2.4, p<0.001). The triage level did not differ, but ED-length of stay was longer in the NSC-group (188 vs. 178 minutes, p = 0.004). Hospitalization was more frequent (84.0 vs. 71.1%, p<0.001) and the length of hospital stay (9 vs. 6 days, p<0.001 was longer in the NSC- than in the SC-group. The number of ED-return visits were comparable between both groups (HR 0.8, 95%CI 0.6–1.1). Mortality within 30-days was higher in the NSC- (20.1%) than in the SC-group (11.0%, HR 1.7 95%CI 1.2–2.4).

**Conclusion:**

Elderly patients present with NSC at the ED regularly. These patients are more often hospitalized and have a substantially higher 30-day mortality than patients with SC.

## Introduction

Elderly patients (≥65 years) use the Emergency Department (ED) more frequently and increasingly over the years, they use more resources and they are more prone to experience adverse health outcomes, like hospitalization, prolonged hospital stay, functional decline and mortality than younger patients [1–5].

“Feeling weak” or ‘being tired’ typifies Non-Specific Complaints (NSC), which are presented by an increasing number of elderly patients who visit the ED [6,7,8]. NSC of these elderly patients arise due to several factors, such as comorbidities, cognitive and functional impairment and communication problems [9,10]. These factors complicate history taking, and lead to a variety of differential diagnoses ranging from social problems to several serious conditions [8,11].

Patients presenting with NSC at the ED often suffer from a serious condition; in one study, serious conditions were present in 59% of patients [8]. Early recognition of both urgency and diagnosis is therefore important to initiate appropriate treatment in a timely fashion. However, patients with NSC are currently not managed by specific protocol, like protocols which are used for patients with specific complaints (SC), as chest pain or severe trauma, because such a protocol is not yet available. The complexity of NSC further leads to misdiagnosis and underestimation of patients with NSC, which influences health outcome in a negative way, such as higher risk of hospitalization and higher in-hospital mortality than other elderly patients [11,12]. The mentioned increased risk of adverse outcomes in elderly patients and presenting with NSC could intensify the risk of adverse outcomes when structured approach lacks [3–5].

Before we can design a structured approach on the ED, we first need to increase our knowledge on these patients with NSC; what are their characteristics, what are their problems, what are their outcomes in daily practice. Real-world knowledge about the patient characteristics, including markers as a proxy for disease, and outcomes of referred patients with NSC is essential to provide insight into patterns of care and improving every day diagnostics and treatment decisions [13].

The objectives of our study were to investigate the characteristics of elderly patients with NSC in daily life and to compare these with patients with SC. In addition, we aimed to investigate whether patients with NSC, compared to patients with SC, have an increased risk of the following outcomes: hospitalization, ED-return visits within 90 days, and 30-day mortality.

## Methods

### Study design, setting and participants

This retrospective cohort study was conducted at the Maxima Medical Centre (MMC), the Netherlands, a 550-bed teaching hospital that has nearly 30,000 ED-visits annually. Of these, 13% are assessed and treated by an internist [14]. In the Netherlands, general practitioners (GPs) refer most patients to the ED after consultation with a specialist, and they both decide whether the patient has to be referred, and to which specific specialty on the ED [15]. Other less common modes of referral are by a medical specialist, self-referral or high urgency ambulance [14]. The acute internist treats patients with problem within the field of general internal medicine, oncology, hematology, nephrology, gastroenterology and rheumatology.

**Fig 1:**
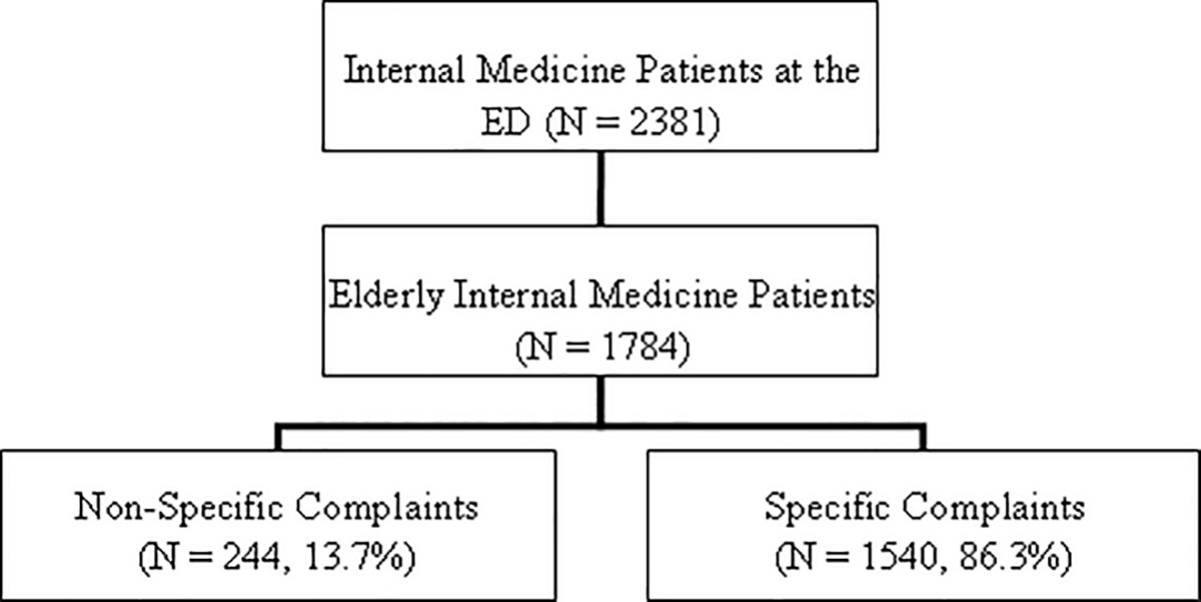
Flow chart of the study population.

Patients were included when they were 65 years or older and when treated by the internist at the ED between the 1^st^ of September 2010 and 31^th^ of August 2011. At the ED, an ED-physician, internist or resident of internal medicine interpreted and documented the referral complaint from the handover information. When the term “Non Specific Complaints” was noted in the patient electronic ED record as the main referral complaint, this was classified as NSC. If NSC were combined with social problems these patients were also classified as NSC. All other main referral complaints documented in the electronic records were classified as SC (Fig 1).

No informed consent was obtained given the retrospective design of the study. Patient privacy was ensured by pseudonymization of the data by replacing all identifying variables and with a unique study patient code. The Institutional Review Board of Máxima Medical Centre approved this study and confirmed that the Medical Research Involving Human Subject Act (WMO) was not applicable.

### Data collection

The premorbid state was retrospectively obtained from electronic patient at the ED: age, gender, medical history, number of used medications, residence and functional status. The following medical data were collected: main referral complaint, cognitive impairment, level of triage, number of problems in history taking, vital parameters (systolic blood pressure, heart rate and temperature) and diagnosis at the moment of discharge from the ED and from the hospital. The following organizational factors were collected: date, time of ED-visit, referral mode, seniority of the physician, number of consultations, number of diagnostic tests (radiology, electrocardiogram, arterial blood gas, laboratory assessment, urine analysis, urine and blood culture), ED-Length of Stay (LOS) and hospital admission. The dates of admission, discharge, last follow-up, ED-return visits, recurrent hospitalizations and of death were collected. All data were extracted by one medical abstractor who was not aware of the study hypothesis.

### Definitions

The Charlson comorbidity index (CCI) was used to quantify the number of comorbidities [16], which were retrieved from the medical records. These records include information from earlier hospital visits and the history taking at the ED. Problems in functional status were defined as the sum of one of the following: recent falls, loss of mobility, problems with communication, weight loss, and pressure ulcers. Cognitive impairment was classified as the presence of one of the following diagnosis in the medical history: delirium, dementia, and confusion. Mode of referral was categorized as via GP, specialist, self-referral or ambulance. Level of triage was based on the Manchester Triage System (MTS), which is routinely used for triage at our ED; the triage groups consisted out of urgent (red and orange), moderate (yellow) and low (green); in our ED, the triage category blue is not used [17]. ED diagnoses and hospital diagnoses categorized according to the ICD-10 [18]. Several categories of the ICD-10 were assembled into the ‘other’ category, which consisted of diseases of the nervous system, musculoskeletal and connective tissue, skin and subcutaneous tissue, eye and adnexa, ear and mastoid, mental, and injury and poisoning. Discharge destination was categorized as home, home care, care home (living at home with additional care), nursing home and other. The ‘other’ category was chosen when patient lived in a rehabilitation centre or a hospice. The ED-LOS was defined as the time, in minutes, between arrival at the ED and leaving the ED [14]. ED-return visits were defined as a revisit of the ED of our own hospital within 90 days.

### Outcome measures

Our first aim was to describe the characteristics of NSC by elderly patients and secondly to compare the primary outcomes between NSC and SC. The outcomes of this study were: hospitalization, in-hospital mortality, 30-day mortality and ED-return visits within 90 days.

### Statistical analysis

All statistical analyses were conducted with SPSS (IBM SPSS Statistics for MacBook, version 22.0, Armonk, New York). Comparisons to evaluate normally distributed differences between NSC- and SC-patients were made using unpaired-t-tests for continuous data and the Chi square test for categorical data. For continuous variables that were not normally distributed, the Wilcoxon-Mann-Whitney-U-Test was used. Missing data were categorized as “missing” and included in the analysis of categorical parameters to explore the influence of missing data. Missing values were excluded when the Chi square test was used. Univariable Cox-regression analysis was performed to investigate the effect of NSC and individual covariates on patient in-hospital mortality, 30-day mortality and 90-day ED-return visit. For each outcome a multivariate Cox-regression analysis was performed to estimate the independent effect of NSC on outcome adjusted for confounders. Confounders were covariates which changed the β-coefficient for NSC on the outcome by more than 10%. Sensitivity analysis was performed to analyze patients who died during hospital admission and the influence on 30-day mortality and ED-return visits. Moreover, sensitivity analysis was performed to analyze the effect of oncology patients on the primary outcomes; patients who had malignancy with and without metastases in the CCI were removed from the analyses. HRs and corresponding 95% confidence intervals (CI) were calculated. A two-sided p-value <0.05 was considered significant.

## Results

In total, 2381 ED-visits were registered for internal medicine of which 1784 (74.9%) were visits by elderly patients. Of these 1784 elderly patients, 244 (13.7%) presented with NSC (Fig 1). The mean age of patients with NSC was 77.6 years (range 65–96) and 46.2% of the patients were male (Table 1). The mean CCI level of these patients was 3, with neoplasm being the most common comorbidity (42.2%).

**Table 1:** The characteristics of the elderly NSC- and SC-patients.

	Total population (N = 1784)	Non-Specific Complaints (N = 244, 13.7%)	Specific Complaints (N = 1540, 86.3%)	P-value
Age, mean (SD)	77.5 (7.7)	77.6 (7.3)	77.5 (7.7)	0.87
Male patients (%)	824 (46.2%)	121 (49.6%)	703 (45.6%)	0.25
Charlson comorbidity index, mean (SD)	2.5 (2.2)	3.0 (2.4)	2.4 (2.2)	<0.001*
Malignancy (%)	551 (30.8%)	103 (42.2%)	448 (29.1%)	<0.001*
Dementia (%)	104 (5.9%)	14 (5.8%)	90 (5.9%)	0.92
Diabetes Mellitus (%)	414 (23.2%)	57 (23.4%)	357 (23.2%)	0.16
Cardiovascular (%)	192 (10.8%	96 (39.3%)	96 (35.9%)	0.35
Cerebrovascular (%)	254 (14.2%)	42 (17.2%)	212 (13.8%)	0.23
Pulmonary (%)	259 (14.5%)	33 (13.5%)	226 (14.7%)	0.52
No. of medications, mean (SD)	6.5 (3.9)	7.0 (4.2)	6.4 (3.9)	0.04*
Residence (%)				0.02*
Independently	187 (10.5%)	33 (13.5%)	154 (10.0%)	
Home care	164 (9.2%)	32 (13.1%)	132 (8.6%)	
Care home	60 (3.4%)	3 (3.5%)	57 (3.7%)	
Nursing home	79 (4.4%)	8 (1.2%)	71 (4.6%)	
Other	44 (2.5%)	9 (3.7%)	35 (2.3%)	
Cognitive impairment (%)	329 (18.4%)	46 (18.9%)	283 (18.4%)	0.85
Functional status (%)				0.04*
1 problem	663 (37.2%)	120 (49.2%)	543 (35.3%)	
>1 problem	232 (13.0%)	57 (23.4%)	175 (11.4%)	
No problem	17 (1.0%)	1 (0.4%)	16 (1.0%)	

ED = Emergency department. SD = Standard deviation. P-values for NSC- vs. SC-patients: using the Chi-square test, unpaired t-test and Mann Whitney U test.

* = P<0.05.

### Characteristics of NSC- compared with SC-patients

Age and gender were comparable between NSC and SC-patients (Table 1). The CCI was higher in (3.0 vs. 2.4, p<0.001) and the comorbidity malignancy was more common in NSC- (42.2%) than in SC-patients (29.1%, p<0.001). The mean number of medications used was higher in the NSC- than in the SC-patients (7.0 vs. 6.4, p = 0.04). The NSC-patients had more problems in the functional status than the SC-patients (2.0 vs. 1.0, p<0.001).

There were no differences in the mode of referral nor in the level of triage between the NSC- and the SC-patients (Table 2). The patients with NSC less often required more than one consultation at the ED than the SC-patients (7.8 vs. 14.1%, p = 0.01) and the ED-LOS was longer in the NSC-group (188 vs. 178 minutes, p<0.004). The number of admissions to an ICU/MCU was equal for both groups (2.5 vs 2.9%, p = 0.67).

**Table 2:** Emergency department characteristics.

	Non-Specific Complaints (N = 244)	Specific Complaints (N = 1540)	P-value
Mode of referral (%)			0.20
General Practitioner	185 (78.7%)	1087 (73.1%)	
Self-referral	25 (10.6%)	177 (11.9%)	
Ambulance	12 (5.1%)	145 (9.8%)	
Specialist	13 (5.5%)	77 (5.2%)	
Level of triage (%)			0.30
Low	85 (34.8%)	518 (33.6%)	
Moderate	138 (56.6%)	823 (53.4%)	
Urgent	20 (8.2%)	188 (12.2%)	
No triage	1 (0.4%)	11 (0.7%)	
No. of problems at presentation, median (IQR)	2 (1–8)	1 (1–9)	0.10
Vital signs, median (IQR)			
Heart rate (min^-1^)	84 (35–180)	83 (40–200)	0.79
Missing (%)	9 (3.7%)	157 (10.2%)	
Systolic blood pressure (mmHg)	135 (118–156)	140 (123–165)	<0.001*
Missing (%)	7 (2.9%)	137 (8.9%)	
Temperature (°C)	37.2 (29–40)	37.3 (31–41)	0.04*
Missing (%)	8 (3.3%)	162 (10.5%)	
No. of diagnostic tests, mean (SD)	3.3 (1.7)	3.1 (1.8)	0.10
No. of patients with >1 consultation (%)	19 (7.8%)	217 (14.1%)	0.03*
ED-LOS in minutes, median (IQR)	188 (23–421)	178 (6–970)	0.004*
ICU/MCU admissions (%)	7 (2.5%)	39 (2.9%)	0.67

ED = Emergency department. ICU = Intensive Care Unit. MCU = Medium Care Unit. SD = Standard deviation. P-values for NSC-patients vs. SC-patients: using the Chi-square test, unpaired t-test and Mann Whitney U test.

* = P< 0.05.

**Fig 2:**
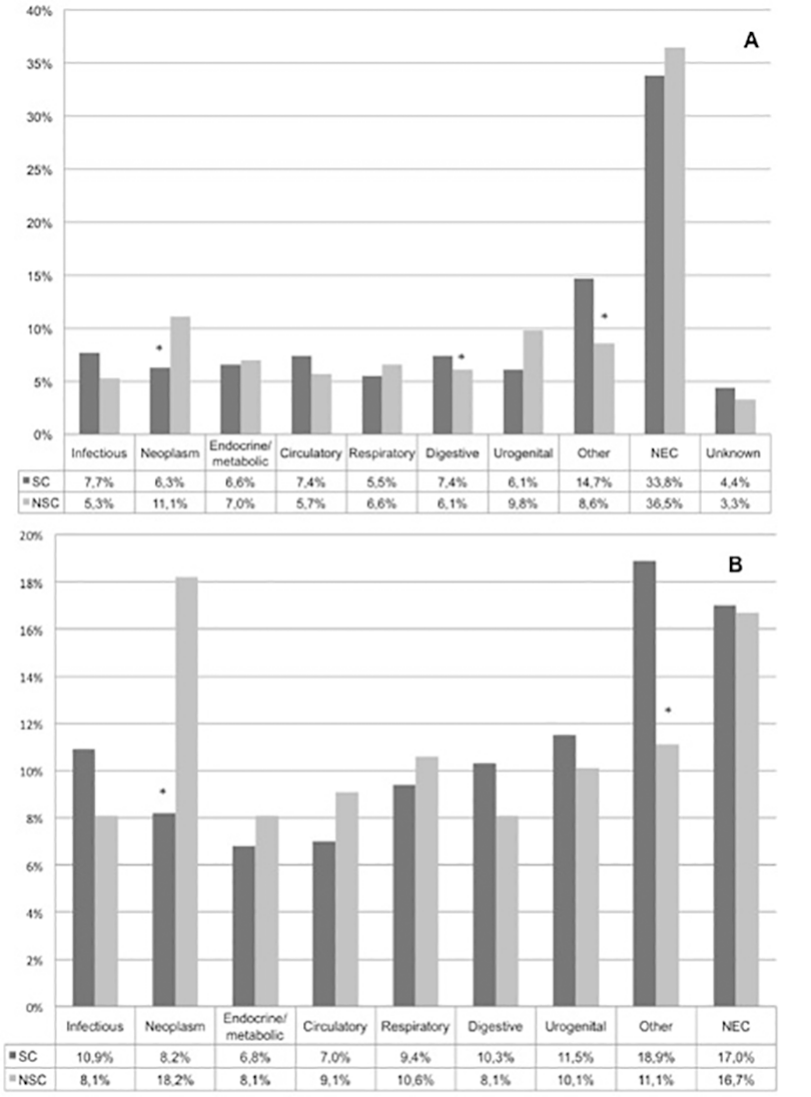
ED discharge diagnosis and hospital discharge diagnosis. A. The diagnosis after workup at the ED and B. diagnosis at discharge from the hospital (NSC = 198 and SC = 1059) (ICD-10 classification). The “other” group includes ICD-10 diseases of the: nervous system, musculoskeletal and connective tissue, skin & subcutaneous tissue, eye and adnexa, ear and mastoid, mental, and injury and poising. NEC = Not Elsewhere Classified. NSC = Non-Specific Complaints. SC = Specific Complaints. * = P<0.05.

The five most common diagnoses at the moment of discharge from the ED (ICD-10 classification) for patients with NSC were not elsewhere classified group (36.5%), neoplasm (11.1%), genitourinary (9.8%), other (8.6%) and respiratory (6.6%) (Fig 2). The largest difference was that patient with NSC were mostly diagnosed with neoplasm compared to the SC-patients (11.1 vs. 6.3%, resp., p<0.001).

### Adverse outcomes for NSC-patients

The NSC patients had a higher hospitalization rate (84.0 vs. 71.1%, p<0.001, HR 1.2 95% CI 1.1 = 0–1.4) and their LOS was longer than that of patients with SC (median 9 vs. 6 days, p<0.001) (Table 3). After hospitalization, an comparable number of NSC-patients and SC-patients returned home (74.0 vs. 68.8%, p = 0.12), but more patients in the NSC-group needed additional home care (28.4 vs. 16.9%, p = 0.01).

**Table 3:** The association between type of complaint and clinical outcomes.

	Non-Specific Complaints (N = 244)	Specific Complaints (N = 1540)	HR (95% CI)
Hospitalization (%)	205 (84.0%)	1095 (71.1%)	1.2 (1.0–1.4)^1^
Length of stay, days, median (IQR)^#^	9 (4–15)*	6 (2–12)*	
In-hospital mortality (%)^#^	32 (15.6%)*	103 (9.4%)*	1.6 (1.1–2.5)^2^
ED-return visits (%)	57 (23.4%)	435 (28.5%)	0.8 (0.6–1.1)^3^
30-day mortality (%)^#^	49 (20.1%)	169 (11.0%)	1.7 (1.2–2.4)^4^

HR = Hazard Ratio. CI = Confidence Interval. ED = Emergency Department.

# = P<0.05.

* fraction of hospitalized patients.

^1^ Multivariate analysis: no confounders.

^2^ Multivariate analysis: ‘CCI’ and ‘prior hospitalization within 90 days’.

^3^ Multivariate analyses: ‘CCI” and prior hospitalization within 90 days’.

^4^ Multivariate analyses: ‘CCI’, ‘number of medications’ and ‘triage level’.

In-hospital mortality for patients with NSC was higher than that of patients with SC (15.6 vs. 9.4%, p<0.001, adjusted HR 1.6, 95%CI 1.1–2.5). Thirty-day mortality was higher as well in patients with NSC than in those with SC (20.1 vs. 11.0%, p<0.001, HR 1.7, 95%CI 1.2–2.4). After multivariable adjustment for confounders (CCI, number of medications and triage level), NSC was still associated with an increased 30-day mortality risk (HR 1.6, 95%CI 1.1–2.2) (Table 3). In addition, sensitivity analysis that excluded the patients who died during hospitalization, showed an increased 30-day mortality risk (unadjusted HR 1.9, 95%CI 1.2–3.0) for patients with NSC. However, after adjusting for confounders ‘CCI’, ‘number of medications’ and ‘triage level’ there was no difference between NSC and SC-patients (HR 1.6, 95% CI 0.9–2.7). Moreover, the sensitivity analyses, which excluded malignancy patients, showed still higher in-hospital mortality and higher 30-day mortality risk among patients with NSC compared to SC-patients (S1 Fig).

Of all patients, 492 (26.7%) revisited the ED within 90 days. The number of ED-return visits was however comparable for NSC- and SC-patients (23.4 vs. 28.2%, resp.; HR 0.8, 95%CI 0.7–1.1), even after adjustment for ‘confounders’ (HR 0.8, 95%CI 0.6–1.0) (Table 3). Another sensitivity analyses, which excluded patients who died during hospitalization, revealed no significant difference (adjusted HR 0.8, 95%CI 0.6–1.1) between NSC- and SC-group either.

## Discussion

Our study provides a descriptive overview of the characteristics and the adverse outcomes of NSC elderly patients who presented as NSC at the ED in comparison with SC-patients. The prevalence of NSC among all elderly patients was 13.7%. These patients with NSC had a higher level of comorbidity (mainly neoplasms), used more medications, were more often hospitalized and had longer hospital-LOS than patients with SC. Most importantly, patients with NSC had a higher 30-day mortality rate. The only adverse outcome that was not different between the two groups was the number of ED-return visits within 90 days.

We found a prevalence of 13.7% of elderly patients with NSC in our ED, which is comparable with that in other studies (13–21.5%) [8,9,19]. However, these studies used different terms and definitions of NSC: one study defined patients as NSC when no initial diagnosis could be made after history taking and physical assessment and another when there was lack of social support and no identification of a main specific complaint [8,9]. Despite the different terms and definitions the studies drew a common conclusion; patients with NSC are under triaged, underestimated and suffer mostly from an acute disease [4,8,12,20,21]. Our study focused on the daily practice of patients with NSC as main referral complaint. Real-world studies are studies who aiming to analyze medical data under real life conditions are necessary to detect additional information on the quality of care [13].

We, like others, found that elderly patients with NSC had more comorbidities and used more medications compared to patients with SC [9,10]. These factors in combination with functional and communication problems may explain the development of NSC, where they complicate the diagnostic process at the ED. The complexity of the diagnostic process of patients with NSC is reflected by the high proportion of incorrect diagnoses that was found in one study (53%) [11]. These misdiagnoses are mostly due to the lack of diagnostic clues and the lack of knowledge of physicians, which complicates a structured evaluation at the ED [6]. Additionally, patients with NSC have a variety of discharge diagnoses [8,11,12]. These factors may delay evaluation at the ED and negatively influence health outcomes [12]. Currently, we are probably not able to adequately diagnose and manage these patients on the ED, as we found that patients with NSC were mostly diagnosed within the not elsewhere classified group (36.5%) at discharge from the ED. On top of that, we found that patients with NSC had an increased risk of adverse outcomes, which underscores the seriousness of the problems of these NSC-patients. Therefore, evaluating the problem of NSC in further research seems to be sensible.

We, like others, found that differences in the organizational factors were different between NSC and SC, as NSC patients have a complex diagnostic process, because they have longer ED-LOS, higher admission rates, and a longer hospital LOS than patients with SC [1,22]. These organizational factors also underscore the complexity of patients with NSC. A structured approach using screening tools might assist in the triaging and evaluation of patients with NSC in combination with medical factors [9]. Current geriatric screening tools for elderly patients that identify the risk of adverse health outcomes, such as the identification of seniors at risk and triage risk screening tool, are unfortunately not very useful due to the low specificity of these tools [23]. Early recognition by means of diagnostic tools combined with medical factors might also improve the logistic factors next to the patient outcomes, however appropriate tools are not yet available.

The higher mortality in NSC patients, which was the main finding of our study, was higher (30-day mortality 20.1%) than in other studies (5.5%-7.5%) [4,8,20]. Despite unawareness of physicians and the late recognition of acute disease [8,9], another explanation of the high mortality rate may be our study population; specifically the high prevalence of malignancy (42.2%). However, after adjustment for this high prevalence of malignancy, 30-day mortality remained higher for patients with NSC than for those with SC. Moreover, the NSC-patients mainly died during hospitalization which have been shown in the sensitivity analyses. It is possible that patients with NSC are patients who are in the terminal stage of chronic disease [4]. The current triage system in our ED possibly underestimates these NSC patients, which could contribute to late recognition of critical illness and high mortality [912]. We investigated different disease markers, such as vital parameters and triage level at the ED. However, we found no relevant differences in these markers. In conclusion, it is unclear which factors lead to this high mortality.

We found that the ED-return visits were not different in our study between NSC and SC. This could be explained by the fact that social/care problems are common in NSC-patients, which often necessitates adjustments in care. Patients are discharged with additional care at home or they are transferred to a care facility to eliminate the risk of a return visit. In line with this, one study concluded that 9.6% of the patients with NSC are discharged from the hospital to a higher level of care [10]. Another explanation for not finding more ED-return visits in NSC-patients is that the high mortality in the NSC-patients “prevented” the revisits. However, this was not shown by our sensitivity analyses. The ED-return visits does not explain vulnerability of patients with NSC. However, all elderly patients were vulnerable as evidenced by the 25% revisit-rate, independent of their complaint being NSC or SC. These findings are in accordance with other studies, and should be considered problematic [10].

Several limitations could have influenced our study results. The main limitation is its’ retrospective single-center design, which may have led to coding errors and missing values. Mistakes in the interpretation of the handover may have influenced the categorization of complaints into NSC and SC. We decided to rely on the first interpretation of the complaint, before history taking and additional tests were performed. This could have led to classifying more patients as having NSC than actually existed. However, this interpretation of the physician–early in the process–was our area of interest, because it has been shown that patients with NSC are mostly low urgent triaged patients who are prone to receive delayed care at the ED [8,12]. In addition, we studied the association of NSC with (amongst others) organizational factors on the ED, and our hypothesis was that having NSC influences these factors immediately after arrival at the ED. Another limitation is that, due to the design of the study we could not rely on validated, state-of-the-art instruments for comprehensive geriatric assessment because they were mostly not mentioned in the patient electronic file. However, the study is a reflection of real practice, and real practice has to deal with missing information, we are used to make decisions based on the information, creativity and experience we have as physicians. Thirdly, the ED-return visits were only measured for our ED; other nearby EDs were not included in the sample. However, patients are mostly referred back to the hospital they have visited before. Lastly, we, in contrast to others, did not exclude patients with SC in their medical history, neither did we exclude patients with fever, hemodynamically instability, nor with terminal conditions [8,24]. This means that we provided a broad clear profile of the patients who were referred as patients with NSC in daily practice.

In conclusion, our real-life study shows that NSC elderly patients presented to the internal medicine ED most often had a history and diagnosis of malignancy, have more medications, they have a longer ED-LOS, less specialist consultations, more hospitalizations, longer hospital-LOS, and higher 30-day mortality than patients with SC. We have shown that NSC might be a predictor of adverse outcome, diagnostic tools are necessary to improve the quality of care for elderly patients with NSC.

## Supporting information

S1 Fig Multivariate analyses of patient outcomes without malignancy patients. HR = Hazard Ratio. CI = Confidence Interval. ED = Emergency Department. # = P<0.05. * = fraction of hospitalized patients. ^1^ Multivariable analyses: no confounders. ^2^ Multivariable analyses: no confounders. ^3^ Multivariable analyses: ‘CCI’ and ‘prior hospitalization within 90-days’. ^4^ Multivariable analyses: ‘CCI’.
(DOC)
