# Supplementary material for: Elderly emergency patients presenting with non-specific complaints: Characteristics and outcomes
Source: PLoS One. 2017 Nov 30;12(11):e0188954. doi: 10.1371/journal.pone.0188954 (PMC5708794; doi:10.1371/journal.pone.0188954)
Supplement: S1 Fig — HR = Hazard Ratio. CI = Confidence Interval. ED = Emergency Department. # = P<0.05. * = fraction of hospitalized patients. 1 Multivariable analyses: no confounders. 2 Multivariable analyses: no confounders. 3 Multivariable analyses: ‘CCI’ and ‘prior hospitalization within 90-days’. 4 Multivariable analyses: ‘CCI’. (DOC) [file pone.0188954.s001.doc]

**Supporting information**

**S1 Table 1. Multivariate analyses of patient outcomes without malignancy patients.**

|  | **Non Specific Complaints (N=139)** | **Specific Complaints (N=1066)** | **HR (95% CI)** |
| --- | --- | --- | --- |
| Hospitalization (%)^#^ | 112 (80.6%) | 736 (69%) | 1.2 (0.96 – 1.4) ^1^ |
| Length of stay, median (range) ^#^ | 9 (4 – 14) ^*^ | 6 (2 – 12) ^*^ |  |
| In-hospital mortality (%) | 14 (12.5%) | 58 (7.9%) | 1.8 (1.0 – 3.3) ^2^ |
| ED-return visits (%) | 29 (20.9%) | 251 (23.8%) | 0.9 (0.6 - 1.3)^3^ |
| 30-day mortality (%)^#^ | 21 (15.1%) | 81 (7.6%) | 2.0 (1.2 – 3.2)^4^ |
| HR = Hazard Ratio. CI = Confidence Interval. ED = Emergency Department. # = P<0.05. * = fraction of hospitalized patients. ^1^ Multivariable analyses: no confounders. ^2^ Multivariable analyses: no confounders. ^3^  Multivariable analyses: ‘CCI’ and ‘prior hospitalization within 90-days’. ^4^ Multivariable analyses: ‘CCI’. | | | |
